# MiR-149-3p promotes the cisplatin resistance and EMT in ovarian cancer through downregulating TIMP2 and CDKN1A

**DOI:** 10.1186/s13048-021-00919-5

**Published:** 2021-11-19

**Authors:** Jin Wang, Lingxia Liu

**Affiliations:** Department of Gynecology, Banan People’s Hospital of Chongqing, No. 659, Yunan Avenue, Banan District, Chongqing, 401320 China

**Keywords:** Ovarian cancer, miR-149-3p, CDKN1A, TIMP2, Cisplatin resistance

## Abstract

**Background:**

Ovarian cancer (OC), a kind of gynecological cancer, is characterized by high mortality rate, with microRNAs (miRNAs) playing essential roles in it. However, the clinical significance of miRNAs and their molecular mechanisms in OC are mostly unknown.

**Methods:**

miR-149-3p expression was predicted through Gene Expression Omnibus (GEO) data in OC and confirmed by q-PCR in various OC cells and tissues from patients with different clinical characteristics. Moreover, its roles in terms of proliferation, migration and invasion were measured by CCK-8, colony formation, wound healing and transwell assays in OC cells including cisplatin-resistant and cisplatin-sensitive cells. And its effect on epithelial-mesenchymal transition was also assessed through detecting related protein expression. Additionally, its potential targets were verified by dual luciferase assay and Ago-RIP assay. Finally, its oncogenic functions were explored in vivo.

**Results:**

In data from GSE79943, GSE131790, and TCGA, miR-149-3p was found to be highly expressed in OC tissues and associated with poor survival. In metastasis and chemoresistant tissues and cisplatin-resistant OC cells, its high expression was confirmed. In terms of tumorigenic effects, miR-149-3p knockdown in cisplatin-resistant OC cells inhibited its cisplatin resistance and other malignant phenotypes, while miR-149-3p overexpression in cisplatin-resistant OC cells led to contrary results. Mechanistically, miR-149-3p targeted 3’UTR of CDKN1A and TIMP2 to function as an oncogenic miRNA.

**Conclusion:**

In brief**,** miR-149-3p promoted cisplatin resistance and EMT in OC by downregulating CDKN1A and TIMP2, which might provide a potential therapeutic target for OC treatment.

**Supplementary Information:**

The online version contains supplementary material available at 10.1186/s13048-021-00919-5.

## Background

The incidence of ovarian cancer (OC) ranks the fourth in gynecological malignancies, and the mortality ranks the second in gynecological cancers [1]. Since 60% ~ 70% of patients are diagnosed with advanced stage, about 80% of patients will relapse in the first five years [2]. Although treatment advances and outcomes for OC have been improving over the past few decades, OC has always been a serious threat to women’s health [3]. Therefore, the identification of predictive biological targets and elucidation of potential mechanisms are very important for OC treatment.

The emergence of microRNAs (miRNAs), special type of non-coding RNAs (ncRNAs), has attracted attention for more than two decades. MiRNAs, consisting of about 20 nucleotides [4], have important regulatory functions. By targeting downstream genes at the 3′-untranslated region (UTR), miRNAs could promote their degeneration or inhibit their translation [5, 6]. MiRNAs are engaged in cell proliferation and carcinogenesis [7–9]. Aberrant expression and distribution of miRNAs are closely associated with the onset and progression of many diseases [10–13]. The discovery and research of miRNAs will bring better prospects for disease treatment.

Numerous studies have shown that the development of many cancers and diseases is associated with miRNAs. miR-21 promoted the cell proliferation and suppressed their apoptosis by regulating PTEN/PI3K/AKT in OC [14]. miR-15a-5p, miR-15b-5p and miR-16-5p had a suppressive effect in neuroblastoma [15]. miR-140-5p and miR-146a were overexpressed in the progression of osteoarthritis [16]. miR-34 family could improve the survival rates of patients with lung adenocarcinoma, and block the metastasis and invasion [17]. miR-4513 was an oncogene in breast cancer via targeting TRIM3 [18]. The functional impact of miRNAs on cancers and diseases has been attracting attention. These miRNAs may provide additional potential diagnostic biomarkers for diseases and promote the development of therapeutic processes.

In this study, we discovered a new up-regulated miRNA in OC data from Gene Expression Omnibus (GEO). High miR-149-3p expression was identified in OC tissues and cells, and strongly associated with bad prognosis. In cisplatin-resistant OC cells, miR-149-3p promoted the proliferation, migration and invasion. Moreover, we found that cyclin-dependent kinase inhibitor 1A (CDKN1A) and metalloproteinase-2 (TIMP-2) were the target genes of miR-149-3p. miR-149-3p could promoted cisplatin resistance by binding with CDKN1A and TIMP2. Our study suggested miR-149-3p may serve as a potential therapeutic target for OC patients.

## Methods

### Clinical samples

In this research, all tissues, including 25 OC tissues, 15 normal ovarian tissues, 15 OC tissues with metastasis, 10 OC without metastasis, 15 pairs of chemosensitive OC tissues and chemoresistant OC tissues were collected from Banan People’s hospital of Chongqing. All fresh samples were intact and confirmed histopathologically. The research program was approved by the Banan People’s hospital of Chongqing ethics committee. Signed informed consent was obtained from all patients and their families.

### Cell culture

Four kinds of human OC cell lines, including SKOV3, cisplatin-resistant SKOV3 (SKOV3/DDP), A2780 and cisplatin-resistant A2780 (A2780/DDP) cells, and a human normal ovarian epithelial cell line HOSEpiC cells, were purchased from Cell Bank Type Culture Collection of Chinese Academy of Sciences (Shanghai, China). These cell lines were cultured in DMEM (Dulbecco’s modified Eagle’s medium) supplemented with 10% FBS (fetal bovine serum) in an incubator with 5% CO_2_ at 37 °C.

### Microarray analysis

The gene expression profile of GSE79943 and GSE131790 on OC and normal ovarian were obtained from the Gene Expression Omnibus (GEO). Then the Venn diagram software was used to analyse the two datasets, and it presented that there was a total of five up-regulated miRNAs including miR-149-3p.

### Prognostic analysis

The data of patients with high miR-149-3p expression and low miR-149-3p expression was gathered from the Cancer Genome Atlas (TCGA), and survival probability was assessed by Kaplan Meier which was commonly used to evaluate the influences of various genes on survival.

### Quantitative-PCR (q-PCR) analysis

Total PCR was isolated from OC cells and OC samples by using a PureLink RNA Mini assay kit (Thermo Fisher Scientific, USA). The PrimeScript RT Reagent assay kit (Thermo Fisher Scientific, USA) was applied for the reverse transcription of target genes. For miRNA, the reverse transcription of obtained RNA to cDNA via a specific miRNA Reverse Transcription kit (Thermo Fisher Scientific, USA). The expression of miRNA or target genes were normalized to the endogenous controls. Each sample was needed three replicate wells. The 2-ΔΔCt method was used to calculated the relative expression. Total synthesized primers were purchased from Sangon Biotech (Shanghai, China) and listed in Supplementary Table 1.

### Transfections of cell lines

The cells were inoculated into 6-well plates with the degree of cell fusion reached 60–80%. MiR-149-3p antagomir, NC antagomir, miR-149-3p mimic and NC mimic were purchased from Ribobio (Guangzhou, China). sh-CDKN1A, sh-TIMP2 and sh-NC individually were loaded into pGPU6 vector (GenePharma, China). Lipo 3000 and RNAiMAX (Invitrogen, USA) were used in transfected experiments, and cells were collected after 48 h. The sequences of all primers were listed in Supplementary Table 2.

### CCK-8 assay

One hundred-microlitre cell suspension (1 × 10^5^ cells/mL) was inoculated into per well of a 96-well plate. When the cells were cultured in an incubator for 24, 48 and 72 h, 10-μL CCK-8 reagent (Sigma, USA) was added into each well, then the plates were incubated at 37 °C for 1 h and the optical density (OD) value was measured at 450 nm.

Cell suspension was inoculated into each well of a 96-well plate at a density of 1 × 10^5^ cells/mL, followed by different concentrations (0, 1, 2, 4, 8 μg/mL) of cisplatin (Selleck Chemicals, USA) treatment for 72 h. Then, the sensitivity of cells to cisplatin was measured by CCK-8.

### Colony formation assay

Two thousand cells were inoculated evenly into each well of a 6-well plate, cultured in an incubator for 2 weeks at 37 °C. Then the colonies were fixed with 4% paraformaldehyde for 25 min and stained with 1% crystal violet for 30 min.

### Wound healing assay

Cells were seeded evenly into per well of a 6-well plate at a high concentration and cultured overnight, 200-μL sterile pipette tips were applied to scraped a wound line on the cell monolayers, the suspended cells were washed off with PBS, and the plate were maintained in an incubator with 5% CO_2_ at 37 °C, the cell monolayers were captured by a microscope at 0 h and 48 h.

### Transwell assay

Coated with or without Matrigel (BD Biosciences), transwell chambers (Corning, USA) with an 8-μm membrane pore in size were used to detect the migration and invasion of OC cells. 2.5 × 10^4^ cells were suspended in serum-free medium and seeded into upper chamber, with bottom chamber filled with medium with 10% FBS. After incubation for 24 h, the cells were fixed with 4% paraformaldehyde, stained with 1% crystal violet, and counted in 10 randomly selected fields.

### Western blot assay

Total proteins were extracted from different cells and samples, and separated by 10% SDS-PAGE. The PVDF membranes were incubated with the different primary antibodies overnight at 4 °C, including CDKN1A (1:1000, 29,475, CST), TIMP2 (1:1000, 57,385, CST), E-cadherin (1:1000, 31,955, CST), N-cadherin (1:1000, 40,615, CST) and Vimentin (1:1000, ab137321, Abcam). On the following day, the membranes were incubated and immersed in the corresponding secondary antibodies, including the HRP-conjugated goat anti-mouse or anti-rabbit IgG antibody. Blotted proteins were visualized by dropping enhanced chemiluminescent solution, and Image J was used to analyze these photos.

### RNA immunoprecipitation (RIP)

A2780/DDP cells were transfected with miR-149-3p mimic or NC mimic for 48 h, then we collected cells from 6-well plates. Ago2 antibody (Abcam, UK) and IgG were used for RNA immunoprecipitation (RIP) with the EZ-Magna RIP kit (Merck, Germany), and the enrichment levels of CDKN1A and TIMP2 were detected by qPCR assay.

### Dual luciferase reporter assay

The *TIMP2* or *CDKN1A* fragments including the predicted binding sites of miR-149-3p as well as the wide-type fragments were cloned into a dual-luciferase vector. The plasmids were co-transfected into OC cells with miR-149-3p mimic or NC mimic. Dual-Luciferase Reporter Assay System (Promega) was used to measure the luciferase activity according to the manufacturer’s instructions.

### Nude mouse models in vivo

The nude mice were purchased from Guangdong Medical Laboratory Animal Center (Guangdong, China) and were raised SPF-rated animal room (Guangzhou, China). A2780/DDP cells were harvested and transplanted into the inguinal subcutis of nude mice. Cisplatin was injected into the abdominal cavity. Subsequently miR-149-3p antagomir or NC antagomir were directly injected into the tumor tissues. The tumor volume was measured every week. After 6 weeks, total mice were anaesthetized with 1% pentobarbital sodium. We measured and recorded the volume and weight of intact tumor tissues. Tumors were dissected and preserved in liquid nitrogen or a refrigerator at − 80 °C for future experiments.

### Statistical analysis

Three and multiple independent experiments were analysed by using One-way ANOVA. Two groups were analysed via using Student’s t-test. Total data were compared by GraphPad Prism 9.0 and exhibited as mean ± standard error of the mean (SEM). The photographs of Western blot were analysed by Image J software. *P* < 0.05 was considered to be statistically significant.

## Results

### miR-149-3p is upregulated in OC and associated with poor clinical outcome

**Fig. 1:**
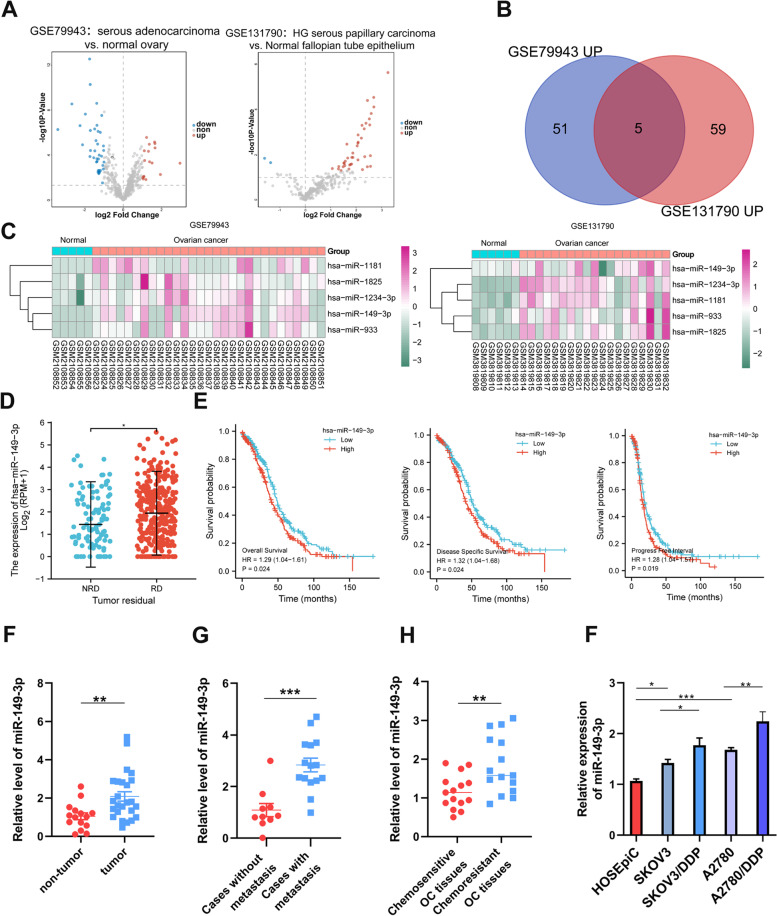
miR-149-3p was overexpressed in OC and associated with poor survival. **A**. The volcano plot of differentially expressed miRNAs in GSE79943 and GSE131790. **B**. Wayne-diagram analysis of up-regulated miRNAs in GSE79943 and GSE131790. **C**. Heatmap of five intersected miRNAs in GSE79943 and GSE131790. **D**. miR-149-3p expression in OC tissues from patients with tumor residual. **E**. Kaplan-Meier analysis was applied to assess the relationship between miR-149-3p and survival. **F**. miR-149-3p expression was detected in OC tissues (*n* = 25) and normal ovarian tissues (*n* = 15) by qPCR. **G**. miR-149-3p expression was detected in cases with metastasis (*n* = 15) or without metastasis (*n* = 10) by qPCR. **H**. miR-149-3p expression was detected in chemosensitive and chemoresistant OC tissues (*n* = 15) by qPCR. **I**. miR-149-3p expression was detected in OC cells by qPCR. *P < 0.05. All experiments were repeated three times with similar results

To identify miRNAs related with the development of OC, firstly, we analyzed the differentially expressed miRNAs from GSE79943 between group serous adenocarcinoma and group normal ovary, as well as from GSE131790 between group HG serous papillary carcinoma and group normal fallopian tube epithelium. Then, we found that miR-149-3p was one of the most up-regulated miRNAs in two datasets (Fig. 1A-C). Moreover, The Cancer Genome Atlas (TCGA) data showed that miR-149-3p was highly expressed in OC with tumor residual compared with non-tumor residual (Fig. 1D). And High expression of miR-149-3p was correlated with poor overall survival, disease specific survival and progress free interval (Fig. 1E). Furthermore, we evaluated the expression of miR-149-3p in clinical OC samples by q-PCR. Compared with the non-tumor tissues, miR-149-3p was significantly upregulated in OC tissues (Fig. 1F). In addition, miR-149-3p was highly expressed in metastatic OC tissues (Fig. 1H). Meanwhile, compared with chemosensitive OC tissues, miR-149-3p was overexpressed in chemoresistant OC tissues (Fig. 1G). We also verified the expression of miR-149-3p in OC cell lines. The results showed compared with immortalized HOSEpiC cells, the expression of miR-149-3p was significantly higher in SKOV3 and A2780 cells, and further upregulated in cisplatin-resistant SKOV3 and A2780 cells (Fig. 1I). Collectively, the above results indicated that up-regulated miR-149-3p correlated with poor survival.

### miR-149-3p inhibitor suppressed cisplatin resistance in cisplatin-resistant OC cells

**Fig. 2:**
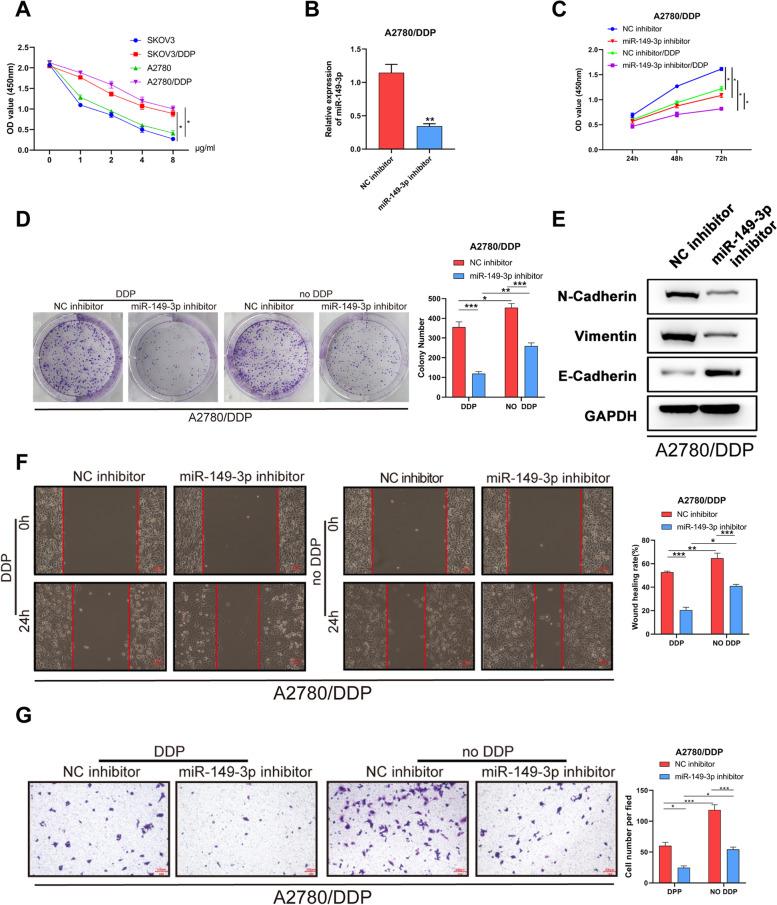
miR-149-3p knockdown inhibited the proliferation, EMT, migration and invasion of cisplatin-resistant OC cells. **A**. The optimal concentration of cisplatin was identified by CCK-8 assay. **B**. miR-149-3p expression was detected in A2780/DDP cells after transfection with miR-149-3p inhibitor or NC inhibitor. **C-D**. The proliferation of A2780/DDP cells was detected by CCK-8 (**C**) and colony formation (**D**) assays. E. Western blot was performed to detect EMT-related protein expression of A2780/DDP cells. **F**. The migration of A2780/DDP cells was detected by Wound healing assay. **G**. The invasion of A2780/DDP cells was detected by transwell assay. *P < 0.05. All experiments were repeated three times with similar results

To investigate the resistance of OC cells to cisplatin, SKOV3, SKOV3/DDP, A2780 and A2780/DDP cells were treated with different concentrations of cisplatin and detected by CCK-8. Then we found the IC50 of cisplatin in SKOV3/DDP and A2780/DDP cells was 2.5 μg/mL (Fig. 2A).

To explore the effects of miR-149-3p on the proliferation, migration and invasion of OC cells, A2780/DDP cells were transfected with miR-149-3p inhibitor, and the transfection efficiency was confirmed by q-PCR (Fig. 2B). The results of CCK-8 and colony formation experiments showed that compared with cells transfected with NC inhibitor, miR-149-3p inhibitor suppressed the proliferation and colony formation of A2780/DDP cells with or without cisplatin treatment (Fig. 2C-D). Besides, we performed Western blot to detect the effect of miR-149-3p on EMT of OC cells. The results showed that miR-149-3p inhibitor induced up-regulated E-Cadherin, down-regulated N-Cadherin and down-regulated Vimentin of A2780/DDP cells (Fig. 2E). Moreover, scratch test and transwell assay revealed that miR-149-3p inhibitor inhibited the migration and invasion of A2780/DDP cells (Fig. 2F-G). These results indicated that miR-149-3p inhibitor suppressed the proliferation, migration, invasion and cisplatin resistance of A2780/DDP cells.

### Overexpressing miR-149-3p reduced cisplatin sensitivity in OC cells

**Fig. 3:**
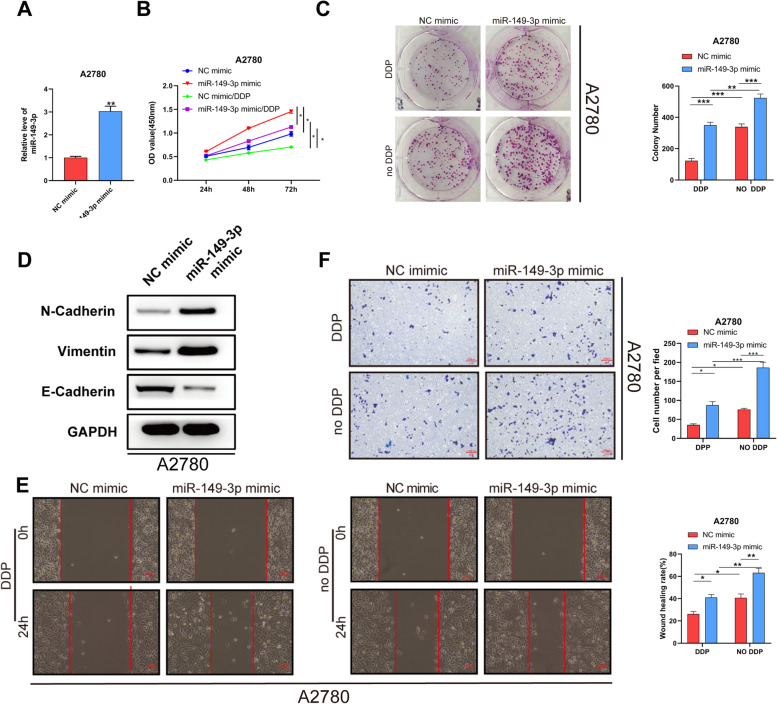
miR-149-3p overexpression promoted the proliferation, EMT, migration and invasion of cisplatin-sensitive OC cells. **A**. miR-149-3p expression in A2780 cells transfected with miR-149-3p mimic or NC mimic. **B-C**. The proliferation of A2780 cells was detected by CCK-8 (**B**) and colony formation **C**) assays. **D**. EMT of A2780 cells was detected by Western blot. **E**. The migration of A2780 cells was detected by Wound healing assay. **F**. The invasion of A2780 cells was detected by transwell. *P < 0.05. All experiments were repeated three times with similar results

In order to study the effects of miR-149-3p on the functions of OC cells, we overexpressed miR-149-3p in A2780 cells, and the transfection efficiency was confirmed by q-PCR (Fig. 3A). Then, the proliferation and colony formation were detected and the results showed that miR-149-3p mimic could promote A2780 cell proliferation with or without cisplatin treatment (Fig. 3B-C). Compared with NC mimic, down-regulated protein expression of E-Cadherin and up-regulated protein expression of N-Cadherin or Vimentin were found in A2780 cells transfected with miR-149-3p mimic (Fig. 3D). The results of scratch test and transwell assay revealed that miR-149-3p mimic strengthen the migration and invasion of A2780 cells (Fig. 3E-F). These results illustrated that miR-149-3p could promoted proliferation, migration, invasion and cisplatin resistance in A2780 cells.

### miR-149-3p inhibited TIMP2 and CDKN1A in OC

**Fig. 4:**
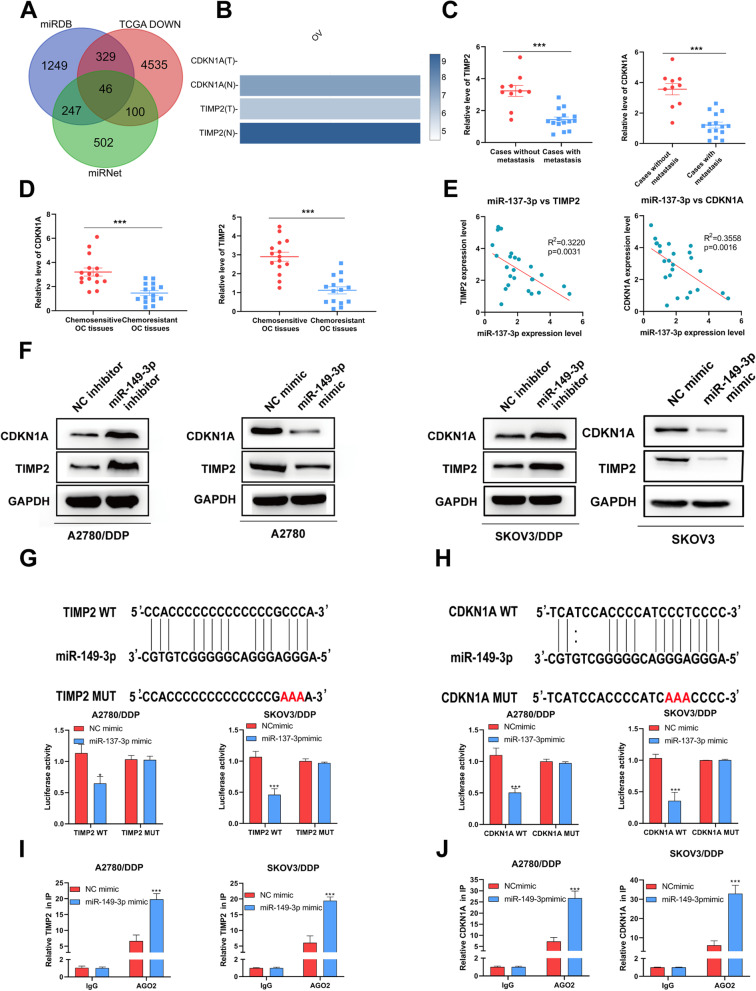
miR-149-3p targeted TIMP2 and CDKN1A. **A**. Target genes of miR-149-3p was predicted. **B**. The expression of TIMP2 and CDKN1A in OC TCGA datasets. **C** The expression of TIMP2 and CDKN1A in cases with metastasis (*n* = 15) or without metastasis (*n* = 10). **D**. The expression of TIMP2 and CDKN1A in chemosensitive and chemoresistant OC tissues (*n* = 15). **E**. Correlation between miR-149-3p expression and TIMP2/CDKN1A expression was determined by Pearson correlation. **F**. q-PCR and Western blot were used to detect the expression of TIMP2 and CDKN1A in cells transfected with miR-149-3p inhibitor or mimic. **G**. The luciferase activities in A2780/DDP cells and SKOV3/DDP cells co-transfected wild-type (WT) or mutant (Mut) *CDKN1A* plasmid together with miR-149-3p mimic or NC mimic. **H**. The luciferase activities in A2780/DDP cells and SKOV3/DDP cells co-transfected wild-type (WT) or mutant (Mut) *TIMP2* plasmid together with miR-149-3p mimic or NC mimic **I-J**. Ago2-RIP assay was used to detect the enrichment of TIMP2 and CDKN1A. *P < 0.05. All experiments were repeated three times with similar results

We than attempted to predict the potential targets of miR-149-3p by analyzing the OC data from TCGA, and found two down-regulated proteins, TIMP2 and CDKN1A (Fig. 4A-B). After that, we evaluated the expression of two proteins in OC samples. We found that TIMP2 and CDKN1A were lowly expressed in cases with metastasis and chemosensitivity (Fig. 4C-D). And the results of q-PCR revealed a negative correlation between miR-149-3p and TIMP2/CDKN1A in 20 OC samples (Fig. 4E). Verifying that, the expression of TIMP2 or CDKN1A were decreased at both gene and protein levels in A2780 and SKOV3 cells transfected with miR-149-3p mimic, and were enhanced in A2780/DDP and SKOV3/DDP cells with miR-149-3p inhibitor (Fig. 4F). Furthermore, dual luciferase assay showed that miR-149-3p mimic inhibited the luciferase activity of the wild-type (Wt) *TIMP2* 3’UTR and *CDKN1A* 3’UTR, but had no effect on the mutant (Mut) sequence of the binding site (Fig. 4G-H). And Ago2-RIP assay showed that miR-149-3p overexpression enhanced the TIMP2/CDKN1A enrichment compared with NC mimic group (Fig. 4I-J). Therefore, it indicated that TIMP2 and CDKN1A were the target genes of miR-149-3p.

### miR-149-3p promoted cisplatin resistance by downregulating TIMP2 and CDKN1A

**Fig. 5:**
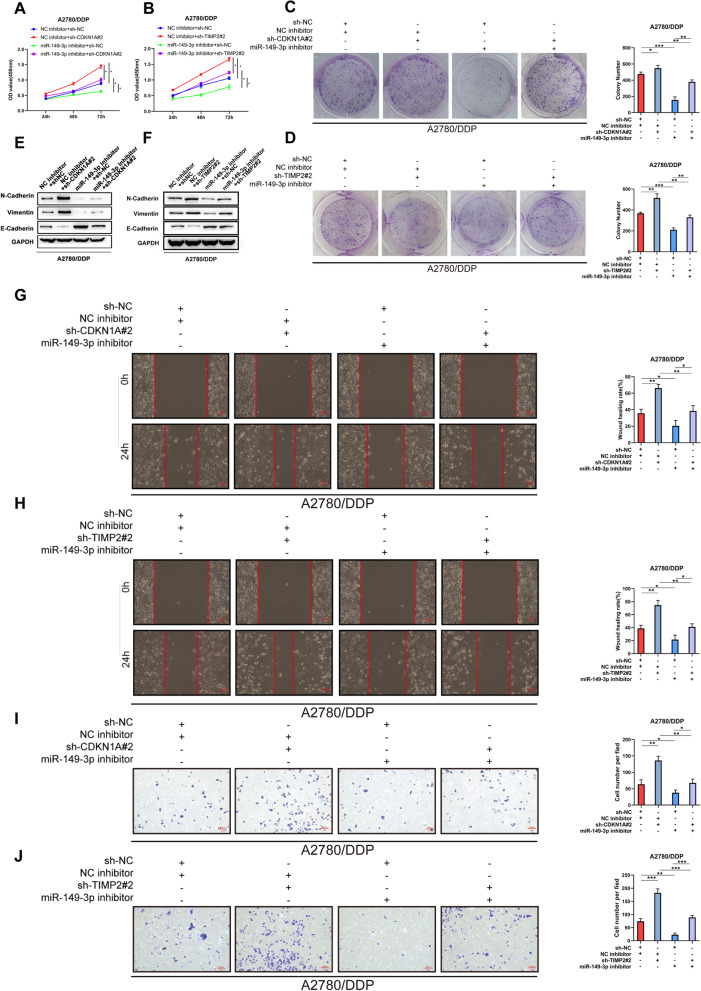
miR-149-3p knockdown rescued the inhibitory effect of TIMP2 and CDKN1A silence. **A-D**. CCK-8 and colony formation assays were performed to detect the proliferation of A2780/DDP cells co-transfected with miR-149-3p inhibitor and sh-CDKN1A#2 (**A-B**) or sh-TIMP2#2. **C-D**. **E-F**. Western blot was performed to detect EMT of A2780/DDP cells co-transfected with miR-149-3p inhibitor and sh-CDKN1A#2 (**E**) or sh-TIMP2#2 (**F**). **G-H**. Wound healing assay was performed to detect the migration of A2780/DDP cells co-transfected with miR-149-3p inhibitor and sh-CDKN1A#2 (**G**) or sh-TIMP2#2 (**H**). **I**. Transwell assay was performed to detect the invasion of A2780/DDP cells co-transfected with miR-149-3p inhibitor and sh-CDKN1A#2 or sh-TIMP2#2. *P < 0.05. All experiments were repeated three times with similar results

To investigate whether miR-149-3p promoted cisplatin resistance in OC cells by decreasing the expression of TIMP2 and CDKN1A, we transfected A2780/DDP cells with miR-149-3p inhibitor, sh-TIMP2, sh-CDKN1A. q-PCR showed that CDKN1A and TIMP2 were significantly down-regulated in cells with sh-CDKN1A#2 or sh-TIMP2#2, which could be rescued by miR-149-3p knockdown (Supplementary Fig. 1A-B). Through CCK-8 and colony formation assays, we found that sh-CDKN1A#2 or sh-TIMP2#2 enhanced the proliferation of A2780/DDP cells after cisplatin treatment (Fig. 5A-D). Besides, sh-CDKN1A#2 and sh-TIMP2#2 promoted EMT, manifested by downregulated E-Cadherin protein expression and upregulated Vimentin and N-Cadherin protein expression (Fig. 5E-F). Moreover, scratch test (Fig. 5G-H) and transwell experiment (Fig. 5I) exhibited that migration and invasion were also increased in A2780/DDP cells after transfection with sh-CDKN1A#2 and sh-TIMP2#2. Importantly, miR-149-3p knockdown can partially suppressed these effects. Taken together, miR-149-3p enhanced the cisplatin resistance of cisplatin-resistant OC cells by downregulating CDKN1A or TIMP2.

### MiR-149-3p enhanced cisplatin resistance in vivo

**Fig. 6:**
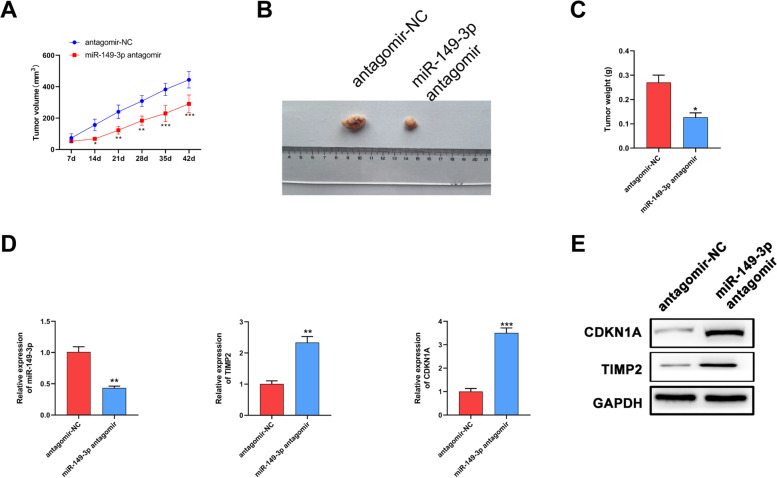
miR-149-3p promoted OC cells tumorigenesis and growth in vivo. **A-C**. A2780/DDP cells were grown in the inguinal subcutaneous region of nude mice to establish tumor growth model. Tumor volume growth curves (**A**), representative images (**B**) and tumor weight (**C**). **D** The mRNA expression of miR-149-3p, TIMP2 and CDKN1A was detected by q-PCR. **E**. Western blot was performed to detect the protein expression of TIMP2 and CDKN1A. *P < 0.05. All experiments were repeated three times with similar results

To further investigate whether the miR-149-3p affect the cisplatin resistance in vivo, we established tumor growth model in nude mice. Compared with NC group, miR-149-3p antagomir significantly inhibited tumor growth, manifested by decreased tumor volume and weight (Fig. 6A-C). Additionally, we used Western blot and q-PCR assays to detect the expression of miR-149-3p, CDKN1A and TIMP2 in tumors. The results showed that after miR-149-3p antagomir injection, miR-149-3p expression was downregulated and CDKN1A and TIMP2 expression were upregulated in tumors (Fig. 6D-E). Therefore, the above data indicated that miR-149-3p might contribute to the tumorigenesis of OC in vivo*.*

## Discussion

Accumulating evidence suggests that abnormal miRNA expression is closely associated with the onset and progression of various diseases. In this study, we found that miR-149-3p was overexpressed in OC and predicted poor survival. miR-149-3p could enhance proliferation and migration by targeting CDKN1A and TIMP2 in vitro, and promote tumor growth and weight in vivo. These findings illustrated that miR-149-3p was an oncogenic gene in OC development and might become a biomarker for OC treatment.

In 2020, the World Health Organization reported that there were 308,060 OC patients worldwide, and the death toll would reach 193,81111 [1]. However, indetectable initial stage, high recurrence rate, and increased drug resistance within 5 years after treatment result in a 5-year survival rate of approximately 47% [19]. Residual tumor is also a very important risk factor and a fatal factor. Patients with few residual tissues will have a high survival rate and benefit prognosis [20]. Physicians have been trying to find better treatment strategies, hoping to detect the occurrence of OC at an early stage, to avoid the regrowth of the tumor as well as to improve the survival rate of patients. In this study, we found that miR-149-3p was upregulated and high expression of miR-149-3p predicted poor prognosis. These results illustrated that miR-149-3p might be an emerging reference indicator and therapeutic target for OC patients.

In recent years, miR-149-3p has been reported to play an important role in various diseases. miR-149-3p was aberrantly expressed in several cancers, including bladder cancer, prostate cancer, breast cancer, lung cancer and endometrial cancer, where miR-149-3p inhibited the activation of DAB2IP (Disabled-2 Interacting Protein) to enhance the cells viability and aggressiveness [21]. In addition, miR-149-3p was involved in the regulation of tumor microenvironment, helping cancer cells escape the surveillance of the immune system by targeting immune proteins [22]. Here, miR-149-3p overexpression enhanced the cisplatin resistance of OC cells. And miR-149-3p knockdown reduced the cisplatin resistance in vivo and in vitro. Therefore, it indicated that decreased expression of miR-149-3p contributed to inhibit the growth and migration of OC cells.

We predicted that CDKN1A and TIMP2 were two potential targets of miR-149-3p by using public data from TCGA. Overexpression of miR-149-3p down-regulated the mRNA and protein expression of both proteins. In contrast, miR-149-3p inhibitor significantly increased the expression of CDKN1A and TIMP2. CDKN1A is a regulatory protein, which plays a crucial role in cell cycle. Abnormal expression of CDKN1A regulates cell cycle process. Some studies showed that up-regulation of CDKN1A led to cell cycle arrest and proliferation inhibition [23–25]. In addition, CDKN1A could suppress cell differentiation by keeping macrophages in a proliferation state [26]. TIMP2 was a kind of tissue inhibitor, which could inhibit tumor growth and invasiveness by restraining the activation of MMPs (matrix metalloproteinases) [27, 28]. In this study, we found that CDKN1A and TIMP2 knockdown could suppress the proliferation, migration and invasion of cisplatin-resistant cells, and these could be rescued by miR-149-3p knockdown.

In conclusion, we found that miR-149-3p was an upregulated miRNA in OC. And high expression of miR-149-3p showed poor prognosis of patients. CDKN1A and TIMP2 were identified as target genes. miR-149-3p downregulated CDKN1A and TIMP2 to promote tumorigenesis and resistance to cisplatin. Our study elucidates the pathogenic mechanism of miR-149-3p and provides a potential target for OC treatment.

## Supplementary Information

**Additional file 1.**

**Additional file 2: Supplementary Table 1.** Sequences of siRNA and shRNA Against Specific Targets. **Supplementary Table 2.** Sequences of PCR primers used in this study.

## Data Availability

The data used to support the findings of this study are available from the corresponding author upon request.
