# Supplementary figures and images for: MiR-149-3p promotes the cisplatin resistance and EMT in ovarian cancer through downregulating TIMP2 and CDKN1A
Source: J Ovarian Res. 2021 Nov 19;14:165. doi: 10.1186/s13048-021-00919-5 (PMC8605569; doi:10.1186/s13048-021-00919-5)

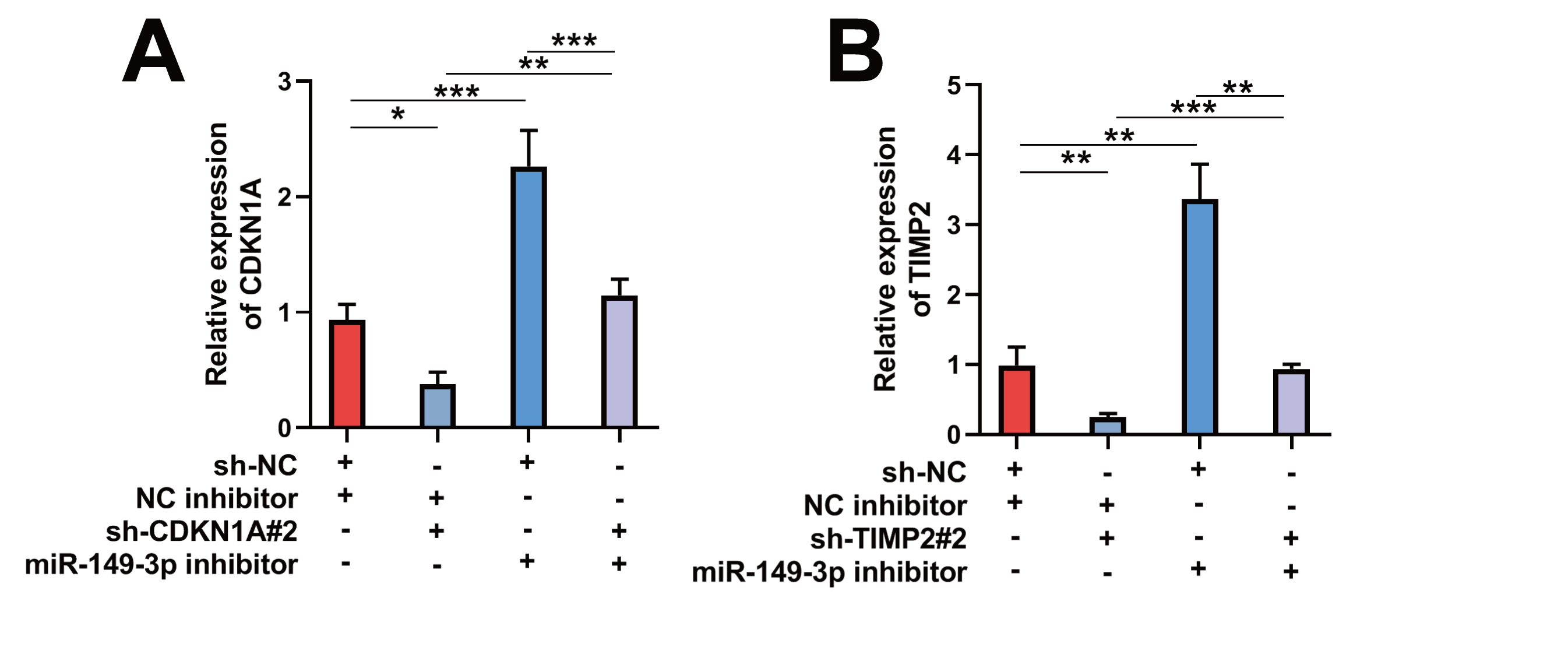

Supplement: Supplementary file 1 — Additional file 1. [file 13048_2021_919_MOESM1_ESM.tif]
