# Supplementary material for: MiR-149-3p promotes the cisplatin resistance and EMT in ovarian cancer through downregulating TIMP2 and CDKN1A
Source: J Ovarian Res. 2021 Nov 19;14:165. doi: 10.1186/s13048-021-00919-5 (PMC8605569; doi:10.1186/s13048-021-00919-5)
Supplement: Supplementary file 2 — Additional file 2: Supplementary Table 1. Sequences of siRNA and shRNA Against Specific Targets. Supplementary Table 2. Sequences of PCR primers used in this study. [file 13048_2021_919_MOESM2_ESM.docx]

**Supplementary Table 1. Sequences of siRNA and shRNA Against Specific Targets**

| sh-TIMP2#1 | 5’-3’ | GGACTCTGGAAACGACATTTA |
| --- | --- | --- |
| sh-TIMP2#2 | 5’-3’ | GGATCCAGTATGAGATCAAGC |
| sh-CDKN1A #1 | 5’-3’ | GAGCGATGGAACTTCGACTTT |
| sh-CDKN1A #2 | 5’-3’ | CCGCGACTGTGATGCGCTAAT |

**Supplementary Table 2. Sequences of PCR primers used in this study**

| TIMP2 | Forward (5’-3’) | GCACATCACCCTCTGTGACT |
| --- | --- | --- |
|  | Reverse (5’-3’) | CTGGTGCCCGTTGATGTTCT |
| CDKN1A | Forward (5’-3’) | CCATGTGGACCTGTCACTGT |
|  | Reverse (5’-3’) | CGGCGTTTGGAGTGGTAGAA |
| miR-149-3p | Forward (5’-3’) | GAACCGGGATGGGAAGTGAC |
|  | Reverse (5’-3’) | GCAAGCGGAACTTCTAGCCT |
| GAPDH | Forward (5’-3’) | TCCAAAATCAAGTGGGGCGA |
|  | Reverse (5’-3’) | TGATGACCCTTTTGGCTCCC |
